# Dimensional Methods Used in the Additive Manufacturing Process

**DOI:** 10.3390/polym15183694

**Published:** 2023-09-07

**Authors:** Ioan Száva, Sorin Vlase, Maria Luminița Scutaru, Zsolt Asztalos, Botond-Pál Gálfi, Adrian Șoica, Simona Șoica

**Affiliations:** 1 Department of Mechanical Engineering, Transylvania University of Brasov, B-dul Eroilor 29, 500036 Brasov, Romania; lscutaru@unitbv.ro (M.L.S.); zsolt.asztalos@unitbv.ro (Z.A.); bpgalfi@unitbv.ro (B.-P.G.); 2 Romanian Academy of Technical Sciences, B-dul Dacia 26, 030167 Bucharest, Romania; 3 Department of Automotive Engineering, Transylvania University of Brasov, B-dul Eroilor 29, 500036 Brasov, Romaniasimonna.soica@unitbv.ro (S.Ș.)

**Keywords:** additive manufacturing, dimensional analysis, geometric analogy, theory of similarity, structural optimization

## Abstract

It is a well-known fact that in the field of modern manufacturing processes, additive manufacturing (AM) offers unexpected opportunities for creativity and rapid development. Compared with classical manufacturing technologies, AM offers the advantages of reducing weight and improving performance and offers excellent design capabilities for prototyping and rapid sample manufacture. To achieve its full potential regarding cost, durability, material consumption, and rigidity, as well as maintaining competitiveness, there are several research directions that have not been explored. One less frequently explored direction is the involvement of dimensional methods in obtaining an optimal and competitive final product. In this review, we intend to discuss the ways in which dimensional methods, such as geometric analogy, similarity theory, and dimensional analysis, are involved in addressing the problems of AM. To the best of our knowledge, it appears that this field of engineering has not fully maximized the advantages of these dimensional methods to date. In this review, we survey mainly polymer-based AM technology. We focus on the design and optimization of highly competitive products obtained using AM and also on the optimization of layer deposition, including their orientation and filling characteristics. With this contribution to the literature, we hope to suggest a fruitful direction for specialists involved in AM to explore the possibilities of modern dimensional analysis.

## 1. Introduction

Nowadays, it is widely acknowledged that AM represents a significant new, fruitful, and promising approach to obtaining low-cost, high-quality, final products within a short period of time, from unique components intended for experimental prototypes to replacement parts for older devices. AM is also a very profitable tool for manufacturing certain high-complexity elements that are intended for use in specialized engineering contexts.

Computer-Aided design (CAD), computer-Aided manufacturing (CAM), and computer numerical control (CNC) offer huge opportunities for obtaining high-quality and highly competitive three-dimensional (3D) objects, but there are significant limitations to the development of an optimized shape and design, which have been discussed in detail elsewhere [1]. The authors of [1] offer an exhaustive analysis of the computer-Aided engineering (CAE) tools involved in AM optimization, from the design stage to the final product shape and load-bearing capacity. As the authors of [1] point out, the design tools involved, i.e., CAD, present several limitations, which they have meticulously analyzed. In addition, they note the significant fact that several engineering and CAD platforms have recently emerged that improve current AM processes, including mesostructured design and optimization programs, along with process management and simulation solutions. Recently designed and developed optimization tools that optimize either shape or topology, which were analyzed in detail in a previous study [1], also contribute to the improvement of traditional AM technologies. In this sense, topological optimization (TO), both continuous and discrete, makes a significant contribution. Starting with a meticulous analysis of the main phases in the AM process, the authors of [1] emphasize the importance of simulation tools in the 3D printing process. These tools prevent the unwanted effects of thermo-mechanical processes or warping, together with stress concentration and other issues. In addition, it is now possible to predict both the geometric deformations and the residual stress state of models using simulation. It should be noted that although simulation tools for plastic materials appeared in 2017, they were applied much earlier in the case of metal AM technologies. Altogether, these process simulations assure better printing technology as well as an optimized final product design. In addition, pre-production tools obtained using the STL-type 3D model offer different solutions for the revision and repair of initial 3D printing files (for example, those drawn from a model defined with polygons to mimic closed surfaces), as well as ensuring that the normal surface to the given surface is correctly oriented. Other useful tools help to optimize print volume, such as layer height and temperature, as well as print speed, i.e., the speed and retraction of the material in fused filament fabrication (FFF) processes, and offer solutions for the necessary support structures.

The authors of [1] mention several publications analyzing TOs [2,3], but relatively few of these studies illustrate their specific application or explore their use when combined with different design tools [4]. As mentioned earlier, in addition to traditional tools, there are now several new and less traditional tools that are exclusively designed and oriented toward additive manufacturing, which contribute to obtaining optimal AM processes.

Since its invention in 1986, rapid prototyping (RP) [5,6,7,8,9,10,11,12,13,14] has enabled the fabrication of complex models and the standardization of their production. The authors of [15] offer a thorough systematization and analysis of AM manufacturing processes, as well as a discussion of the raw materials involved, such as metals, ceramics, composites, and thermoplastics. These materials are destined for use in various applications [16,17,18]. As mentioned by the authors of [19], polymers are the most widely used raw material in *AM* processes. By adjusting the infill density and using structural optimization, parts with complex geometry can be produced without requiring expensive tooling [20].

CAD models enable the production of parts with complex shapes, as well as offering reduced machining requirements and space utilization. It is well-known that AM processes do not require the use of jigs, fixtures, tools, or molds or processes such as milling, injection molding, drilling, or broaching [21]. In addition, the manufacturing process is relatively simple, with minimal wastage comprising only the support materials and loose powders. Such processes have several advantages compared with conventional manufacturing processes; however, their applications in building parts for use as functional products or components are limited [22]. According to the ASTM F2792-12A [23], AM processes can be divided into the following major groups:

1. Binder jetting, involving the powder bed and inkjet head;
2. Directed energy deposition using laser metal deposition (LMD);
3. Material extrusion using FFF;
4. Material jetting using multi-jet modeling (MJM);
5. Powder bed fusion using selective laser sintering (SLS), direct metal laser sintering (DMLS), and electron beam melting (EBM);
6. Sheet lamination, involving laminated object manufacturing (LOM) and ultrasonic consolidation;
7. Vat photo-polymerization using digital light processing (DLP) and the stereo-lithography apparatus (SLA) [24].

Usually, with CAD models, layer-by-layer deposition is used to produce the desired parts. Compared with classical manufacturing processes, AM products cannot meet different functional requirements, such as mechanical, thermal, and electrical properties, thermal stability, dimensional accuracy, surface quality, or multi-axial load-bearing capacity. Furthermore, parts made using AM processes are often rigid or are not able to respond to environmental stimuli [15].

As pointed out by the authors of [25], fused filament fabrication FFF, which uses thermoplastic polymer filaments, is one of the most widely applied and relatively inexpensive AM processes. Naturally, the properties of the filament materials involved significantly define the final product properties, such as strength, geometrical stability, thermal behavior, and electrical conductivity. The limitations of FFF include poor mechanical properties of final products, limited raw materials, restricted part size, and low production rates. In addition, the selected process parameters, filament materials, and material properties have a significant influence on the resulting products.

By applying process parameter optimization, FFF has become more efficient [26,27]. Of course, even with the optimal combination of process parameters established using process parameter analysis, its limitations can only be improved to a certain extent, according to the initial properties of the raw materials [28]. Additionally, not all filament materials, built shapes, or equipment will support a given level of optimization [29].

The authors of [15] also reviewed four-dimensional (4D) printing, which represents a revolutionary advancement in AM processes, mainly regarding the production of parts manufactured using smart materials [30]. So-called 4D printing applies the same techniques as those used in 3D printing; however, depending on the shape memory of the raw materials involved, 4D printing can be used to manufacture new, intelligent, and more complex parts [18,31,32].

The above-mentioned technologies have been applied not only to obtaining objects from plastic materials but also combined metal–plastic objects, as well as objects made only from metal [33,34,35,36,37,38,39,40,41,42,43,44]. In another study [15], a meticulous analysis is performed on filament materials used in FFF, which represents one of the most popular AM processes. One of the earliest technological advances in the field was fuse deposition modeling (FDM), which comprises a simple extrusion process. During FDM, the preheated material, which flows through a nozzle, is deposited layer-by-layer in response to the combined movements of the nozzle and the structure’s platform [45,46,47,48,49,50,51,52,53]. This kind of technology is the main focus of this review.

In addition, we analyze the following technologies, which are applied mainly to metal deposition.

The earlier approaches mentioned above were followed by powder bed fusion processes using different printing techniques, such as direct metal laser sintering (DMLS), EBM, selective heat sintering (SHS), selective laser melting (SLM), and selective laser sintering (SLS), which have been widely analyzed in the literature [54,55,56,57,58,59,60,61,62,63,64,65,66,67,68,69,70,71,72,73,74,75,76,77,78,79,80,81,82,83,84,85,86].

Other printing technologies followed, such as the so-called sheet lamination processes, including ultrasonic additive manufacturing (UAM) and LOM [72,74,84,87,88,89,90,91,92,93]. UAM uses sheets or ribbons of metal, bonded together using ultrasonic welding, whereas LOM, instead of welding, uses layer-by-layer glued-paper deposition to form objects.

Other modern approaches include directed energy deposition (DED) and automated fiber placement (AFP) techniques [94,95,96,97,98,99,100,101,102], which have not yet been considered as a subject of investigation.

Stereolithography (SLA) or photopolymerization (comprising optical fabrication, photo-solidification, and resin printing) is a rapid manufacturing method that uses 3D printing technology for prototyping. During this technique, a photochemical method is used to obtain a three-dimensional object. Stereography is used most frequently in medical engineering; it is an extremely rapid method that can realize complex projects, but its cost is very high. Stereolithography is a very important technique that is used for specifically oriented, high-end applications, but it produces complex models. Therefore, in this review, we instead analyze methods with more wide-ranging engineering applicability.

By addressing significant problems, dimensional methods represent very useful tools for improving the limitations of previous methods. In the next section, we present a brief survey.

In order to obtain useful information on a given structure, hereafter: *prototype*, mathematicians and engineers have developed several easy and inexpensive specialist approaches that allow firm correlations to be drawn using a reduced-scale structure, hereafter: model [103,104,105,106,107,108]. When the results of experimental measurements, performed strictly using an attached model, are transferred to a prototype, it is possible to use these correlations to predict its behavior. This set of correlations, which contains strictly dimensionless variables, represents the so-called model law (ML) [109,110,111,112,113,114,115,116].

Depending on the number of monitored variables and their dimensionless correspondent (tally), correlations can be obtained for relatively simple cases using geometric analogy (GA) and the theory of similarity (TS), respectively, while for very complex phenomena, classical dimensional analysis (CDA) can be used [42,43,46,117,118,119,120,121,122,123,124,125,126,127]. Of course, the associated model must be related to the analyzed prototype as closely as possible to allow for accurate, easy, reproducible, and low-cost experimental investigations. The results obtained using the model will predict prototype behavior under given conditions, which must completely comply with the deduced ML.

TS is suitable for more complex scenarios, where, in addition to structural similarity (which is mainly realized using geometrical similarity), functional similarity is also required. Functional similarity assumes that in both systems, there are similar processes that occur at similar times. Consequently, all physical properties of the analyzed process should exhibit a certain similarity. In this case, at homologous times and homologous points, a given phenomenon occurs, where each η variable is described by $S_{\eta }\left[-\right]=\frac{\eta_{2}}{\eta_{1}}$ as a constant ratio of the values corresponding to the model ($\eta_{2}$) and prototype ($\eta_{1}$). These $S_{\eta }$ dimensionless ratios, hereafter: *scale factors*, are always constant in time and space for a given phenomenon. Of course, the number of scale factors is identical to the number of involved variables when describing an analyzed phenomenon.

To describe a given phenomenon, instead of using the mathematical solution of a complicated equation, some relatively simple correlations between a limited (*n*) number of $\pi_{j},\;j=1\ldots n$ dimensionless variables, which constitute the ML, are applied. In the case of TS, these dimensionless variables, which are obtained using a suitable grouping of terms in an equation, are then replaced with corresponding scale factors, providing the required ML.

Based on the ML thus obtained, the experimental results of the model will provide a prediction of the prototype and the measurement number, as is required for a complete analysis.

In relatively simple scenarios, when GA is applied, the geometric similarity between the prototype and the attached model is compulsory. Geometric similarity assumes a rigorous proportionality of lengths and the respective angular equality for the model and prototype. Consequently, homologous points, lines, surfaces, and volumes of the prototype and model can be defined. Such an imposed similarity assumes a relatively limited flexibility of a model in relation to a prototype.

When the number of these dimensionless variables, $\pi_{j},\;j=1\ldots n$ becomes very large (for more complex phenomena), it is necessary to apply CDA [68,88,94,105,128,129,130,131,132,133,134,135,136,137,138,139,140,141,142,143,144,145,146,147,148].

CDA assures a relatively easy approach for addressing complicated phenomena. It is not intended to replace experimental investigations; rather, it serves to simplify and correct the experimental strategy optimization using the involved $\pi_{j},\;j=1\ldots n$ dimensionless variables, as defined by Buckingham’s π theorem.

To obtain the required set of dimensionless variables, CDA uses three main methods, namely,

- The direct application of Buckingham’s π theorem;

- The application of partial differential equations to the fundamental differential relations of the analyzed phenomenon; using a suitable grouping, the initial variables become dimensionless quantities;
- The transformation of complete, but the simplest, equation(s) related to the phenomenon into dimensionless forms, finally yielding the desired $\pi_{j}$ groups.

A detailed analysis of CDA is included in the references mentioned above. Unfortunately, the CDA method has several shortcomings, including the following:

- Obtaining the desired set of dimensionless variables is rather chaotic, arbitrary, and strongly dependent on the experience and ingenuity of the involved specialist.
- The specialist involved in the ML deduction must possess solid knowledge, both in the field of the analyzed phenomenon and higher mathematics.
- The complete ML is rarely (or occasionally) obtained, mainly due to the limited number of mathematical relations involved that describe the phenomena.
- CDA is not an easy method to master for regular engineers who are involved in prototype–model correlation analysis.

The approach developed by Szirtes [149,150], hereafter: modern dimensional analysis (MDA), solves practically all the shortcomings of CDA. MDA represents a unitary, simple, and particularly accessible methodology with the following advantages:

- The specialist involved can be a regular engineer, without a profound knowledge of the field of the analyzed phenomena. The specialist only needs to review the involved variables, together with their dimensions, which have (or can present) a certain influence on the analyzed phenomena.
- Using the unitary protocol, all insignificant/irrelevant variables are automatically eliminated.
- The approach allows for providing a complete set of the $\pi_{j},\;j=1\ldots n$ dimensionless variables in all cases. Consequently, the complete *ML* is available, which the aforementioned methods/approaches can only produce in particular cases.
- The obtained ML is very flexible, allowing for easy deduction of several useful simplified case studies, which can be associated with particular approaches to the phenomena.
- MDA assures a priori choosing/setting of variables that are directly related to conceived experimental investigations with the model; these variables are hereafter referred to as independent variables.
- The set of independent variables defines the most suitable model that can be associated with a given prototype, thus obtaining the most simple, safe, repeatable, and low-cost testing conditions for the given model in experimental investigations. These variables are freely chosen on an a priori basis for both the prototype and the model.
- The remaining variables of the prototype, hereafter referred to as dependent variables, can only be chosen on an a priori basis. For the model, the dependent variables can be obtained strictly by applying a given (suitable) element of the ML.
- A few of the prototype’s dependent variables are included that cannot be obtained more easily at low cost or by using accessible experimental measurements.
- Consequently, these aforementioned prototype variables must be obtained by applying certain elements of the ML. In fact, their deduction with the help of the ML represents the major goal of the proposed dimensional analysis.
- In addition, when applying MDA, the geometrical analogy between the prototype and the model is compulsory, e.g., the shape of the cross-section in the model can be different from that of the prototype.
- For example, by choosing the material as an independent variable, one can choose different materials for the prototype and the model.

Some of these capabilities of MDA were illustrated in previous works [94,97,151,152].

In the next section, we analyze the ways in which these dimensional methods are involved in different AM problems.

## 2. The Involvement of Dimensional Methods in Additive Manufacturing

From the very large body of literature analyzing the behaviors of polymers, certain studies discussing AM can be highlighted [14,47,50,51,52,53,63,75,79,98,100,102,153,154,155].

### 2.1. The Dimensional Analysis Conceptual Modeling (DACM) Framework

The most appropriate dimensional approach for efficiently establishing a set of dimensionless variables, bearing in mind Szirtes’ theory [149,150], is the dimensional analysis conceptual modeling (DACM) framework developed by Coatanéa [156,157,158,159,160,161,162,163,164,165,166,167]. The DACM provides the following main results.

In his Ph.D. thesis [157,159], Coatanéa proposed a new approach, which he called conceptual modeling of life-cycle design, subsequently perfecting and renaming it the conceptual modeling and simulation framework for system design [158,159,160,161,162]. Currently, modeling and simulation are widely applied to achieve a more efficient final product using AM. A significant issue that needed improvement is the integration of different models and their details. Using the approach developed by Coatanéa, it became possible to increase not only the ability to predict the involved parameters and performance of the desired components in AM but also to improve, step-by-step, the design of the complete manufacturing process. By applying DACM, useful causal graphs integrating AM equipment, as well as the desired components to be printed, can be obtained.

This approach ensures the opportunity to integrate current knowledge into an existing and efficient model. In this context, using useful computer tools, the framework provides the integrated model with an efficient set of governing equations, i.e., the relationships among influencing variables, together with a reduction in the number of aggregate indicators. The obtained correlations will ensure the continuous improvement of the parameters involved and, thus, better machine design, an optimized printable component, a highly accurate 3D-printing process, and efficient control of the manufacturing process. When applied to fused deposition fabrication powder bed fusion technology, this approach leads to an efficient and early redesig, not only of the required component to be obtained but also of the involved AM equipment, along with its control parameters [160,161,162,163,164,165,166,167].

The authors of references [147,148] present similar and equally efficient approaches.

### 2.2. Data-Driven Approaches to Dimensionless Quantities Based on the Experimental Results

Working from the starting point of CDA, the authors of [145] proposed an approach known as dimensionless learning, comprising a two-level machine learning principle for achieving a relatively automatic identification of the involved dimensionless numbers and the corresponding MLs. This approach proved to be very useful, mainly for obtaining a set of dimensionless variables for highly multi-variable systems with few governing equations. Using this methodology, the authors were able to both reduce the number of variables involved and achieve optimization of the analyzed system. They also illustrated its efficiency with several practical examples in the field of 3D printing, using it to diminish porosity formation, and suggested combining the methodology with more data-driven methods.

In an earlier study [128], the authors modeled a flexible barrier in order to manufacture models using 3D printing (i.e., using AM). For this purpose, they applied Buckingham’s π theorem to establish the involved dimensionless numbers and the necessary ML concerning the mechanical aspects of the barriers. They validated the obtained ML using several meticulous experimental investigations. This is a relevant example of the ways in which partial mechanical similitude can be involved in a complex fluid mechanics problem.

Starting with the actual limitations of AM 3D printers, the authors of [36] designed and manufactured a bench-top powder melt extrusion (PME) 3D printer head could print parts directly from powder-based materials rather than filament. They performed a comparative performance analysis of their printer with traditional FFF printers and found that objects printed with their system had comparable visco-elastic properties to those obtained using a classical FFF system. Based on accurate dimensional analysis, their system currently presents a lower resolution. However, this can be improved in the future, which is worthwhile because their approach is very promising. In addition, the relatively significant number of mathematical modeling proposals should also be noted, the details of which are synthesized elsewhere [44,60,62,64,74,138,168,169,170,171,172,173,174,175,176].

The authors of [53] presented some useful results for improving AM using hybrid manufacturing technology. Taking into consideration the shortcomings of classical AM, such as inadequate dimensional accuracy and poor surface quality, the authors combined this method with an additional chipping (milling) machining step, i.e., by removing scrapings from the material, and obtained promising results. The involved materials comprised commercial carbon fiber-reinforced PLA and normal PLA filaments.

One author’s PhD thesis [53] analyzed AM with FDM involving nylon and carbon fiber-reinforced materials. Different samples with different fill patterns were printed and tested, and the results indicated isotropic behavior in the fabricated samples. Based on the results of tensile and three-point bending tests, the influence of strain rate and aging on the samples consistently influenced the sigma–epsilon plots. The purpose of the study was to evaluate the elastic properties of nylon and fiber-reinforced 3D-printed structures according to ASTM standards [23]. To find the Poisson’s ratio and shear modulus of each sample, the author performed a post-processing analysis based on DIC measurement data.

Elsewhere, a similarity study (prototype–model) was performed to optimize an analyzed product. In their contribution [8], the authors conducted a useful survey on AM technologies and their applications in China.

The authors of [62] combined the advantages of AM and TO. They performed an optimization of a car door hinge based on a previous FE simulation. It is well known that AM produces products with complex geometry at very low cost in innovative and high-efficiency conditions. By combining these advantages with those of the optimization afforded by TO, one can assure efficient material redistribution while taking into consideration the imposed design objectives. Starting from the given structural analysis results, the authors performed a topological optimization in order to obtain efficient material redistribution, which was related to the maximal load-bearing capacity of the final product.

Although the control of melt pool geometry using feedback or feed-forward methods is a possibility, the time required for changes in process parameters to translate into adjustments to melt pool geometry is one of the most critical concerns. A second method is to implement multi-physics simulation models, which can provide a good estimate for time process parameter evaluation.

Unfortunately, such models are almost computationally intractable when using an optimization framework to find the process parameters to achieve the desired melt pool geometry as a function of time.

The authors of [60] offered an efficient solution using an original hybrid framework. In their paper, they used machine learning-assisted process modeling and optimization to control melt pool geometry during the building process. They validated the proposed approach using accurate experimental observations. The authors used a tailored 3D analytical model to predict thermal distribution in a moving melt pool.

The obtained results demonstrate the efficiency of model-based optimization using machine learning tools in a data-driven setting and reliable a priori estimates of process parameter evolution, which ensure that the desired melt pool dimensions are obtained for the entire building process. Fused deposition modeling is one of the most commonly used *AM* technologies. One of the main obstacles to obtaining high-quality components with the appropriate mechanical qualities (that is, tensile strength and flexural strength)is the selection of processing parameters.

The authors of [85] experimentally analyzed the influence of the most significant process parameters, i.e., air gap, raster angle, print orientation, layer height, and raster width, on the tensile strength of printed articles patterned using fused deposition. Using an analysis of variance, they measured and evaluated the impact of each parameter involved. The authors used an adaptive neuro-fuzzy technique combined with an artificial neural network to predict the system’s response. Based on a precise analysis of 46 experimental trials, they demonstrated that grid width, air gap, and grid angle have a significant impact on tensile strength.

The predicted tensile strength, which was based on the adaptive neuro-fuzzy approach and an artificial neural network, was in good agreement with the literature. However, fabricating and repairing thin-walled structures using a directed energy deposition process demands a deeper understanding of the properties of the method’s basic building block: clad formation.

The authors of [136] used a multi-scale analytical methodology to predict the mechanical properties of products made from continuous fiber materials using the fused deposition fabrication technique.

Starting their analysis with a micro-scale study (using several micro-models), they obtained the basic properties of individual printed filaments. Afterward, the effect of voids was analyzed on a mesoscale. By proposing an original concentric cylinder model approach, it was possible to evaluate the effect of the voids when a transversely isotropic matrix was considered. By combining plies in different orientations, based on classical laminate theory, they finally established the macro-scale properties of the analyzed materials. A large-number experiment validated the proposed theoretical approach.

The authors of another study [133] applied TS to replace classical experimental investigations on full-scale prototypes with those performed on reduced-scale models to recreate standard structural members with a rectangular hollow structural section (HSS), obtained using AM.

The authors analyzed several simple small-scale supported rectangular HSS beams created using AM and established a generic similarity relationship with a full-scale prototype. Among the most important parameters analyzed were the printing time and the material required for each sample, obtaining useful scaling curves for their manufacture. In addition, using three-point bending tests, the authors obtained scaling curves for the specimens, which were useful for predicting the ultimate load of a full-scale structure.

In order to validate their approach, they performed *FE* analysis with ABAQUS, comparing the theoretical results on load-deflection behavior with those predicted using the scaling curve formula. Their proposed methodology represents a useful and novel approach for achieving feasible and cost-effective modeling of *HSS* beams as well as conceiving new materials and manufacturing techniques. Their additional goal consisted of infill pattern and printing direction analysis, in order to improve the structural behavior of printed models.

The authors of [138] considered the fact that AM process parameters regarding fused filament fabrication represent key factors in the mechanical performance of the resulting components. Thus, they proposed a three-zone model based on the printing pattern, which was realized using different materials for the cover, contour, and inner parts of the model. The authors performed bending tests for validation considering mono-axial tensile test characterization for the first two zones and computer homogenization for the third. Using dimensional analysis, they deduced a model law that correlated the raw and the 3D-printed material mechanical properties based on a reduced number of dimensionless variables. They also analyzed the inter-layer adhesion performance of the three kinds of materials used in their study. Based on this approach, the authors proved that the number of required testing configurations could be diminished by two-thirds, thereby resulting in considerable cost savings.

Based on an approximate solution to energy balance, the authors of [51] developed a dimensionless approach (and a corresponding model) related to the correlation between the dimensionless fiber feed rate (using the Péclet number) and the dimensionless temperature needed for polymer-based 3D printers.

They aimed to establish an allowable feed rate for three polymers used in the 3D printing of various components. In the first test, all polymers showed the same behavior, which was described using a single characteristic/dimensionless curve. Conversely, when they analyzed the passage of the molten polymer through a given small nozzle, another parameter became important, i.e., the diameter of the nozzle. By modifying the expression of the Péclet number, it was possible to account for the hydrodynamic peculiarities in the process and finally obtain a dimensionless curve that was suitable for all diameters and polymers. The obtained results represent useful tools for future work, both for designers when redesigning the extrusion die and when choosing the most suitable polymer for a given final product.

Two other topics are also of interest in this literature review.

In one study [29], the authors analyzed the accuracy of a surgical workflow model for oral implant placement using guides manufactured with SLA and FDM from a biodegradable and sterilizable biopolymer filament. The authors performed an accurate and very promising statistical analysis using a Tukey test.

In another paper [177], the authors performed a detailed comparative analysis of 3D-printed safety protection devices (respirator masks), manufactured using FDM and droplet-based precision extrusion deposition (db-PED) from common food packaging materials. The main issues in their comparative analysis were the mechanical resistance and resistance to dissolution, both before and after the cleaning and disinfection phases, biological safety (cell adhesion and viability), and surface roughness evaluation regarding the accumulation of bacteria and viruses.

Their comparative analysis included comparing home-grade printed masks with industrial-grade masks, with very promising results. In addition, they developed novel approaches for the AM post-processing phases in order to assure human safety when using custom-printed medical devices. They also performed a statistical analysis with a Tukey multiple comparison post hoc test, which yielded very promising results.

### 2.3. Dimensional Approaches to Metal Deposition and Metal-Infused Thermoplastics Using AM

In an earlier study [130], clads obtained by depositing stainless steel 316L (SS316L) powder under three different process parameters (laser power, laser traverse speed, and powder mass flow rate) were investigated, which were found to ensure high repeatability. The authors demonstrated that laser power was the most significant factor for clad depth, but it had little influence on clad thickness. The laser traverse speed was the dominant parameter for clad height, and the powder mass flow rate tended to compensate for depth reduction with thickness gain, resulting in no noticeable effect on clad height.

The authors of [120] analyzed the conductive structures that can be manufactured using 3D printing, i.e., using AM with FDM. They performed an accurate analysis in order to achieve better (i.e., isotropic) electrical properties instead of the actual anisotropic properties.

They started by analytically modeling the electrical conduction of these specially manufactured components and then eventually used finite element (FE) analysis and a dimensional approach. In this way, the authors defined several dimensionless numbers, such as inter-traxel resistance, the anisotropy ratio (which expresses the material effect), the aspect ratio (which expresses the geometry effect), and the number of traxels and meanders (related to discrete print design, thereby determining the total path length). The dimensional approach did not involve the classical Buckingham’s π theorem, only a particular grouping of the involved quantities in the obtained analytical relationships/equations. The obtained results allowed the authors to propose a new, very sensitive tensile and compression sensor, which offered several advantages.

Several authors have observed that continuously increasing laser power is the most effective way to prevent clads from forming with zero dilution, which can serve as an indicator of how well a printed clad is bonded to the substrate. Starting with a set of SS316L clads, they performed dimensionless analysis and proved that this can facilitate the selection of process parameters to meet the given requirements for clad dimensions. Their results represent an improved standard pre-printing tool for use in this process.

The authors of [97] performed a useful dimensional analysis of the SLM process based on the normalization of a relevant energy equation. They identified four significant dimensionless variables including the welding parameter, the melting parameter, vaporization efficiency, and the track size parameter, which, together, are fully able to characterize the thermo-dynamic behaviors seen in the SLM process. Then, the authors carefully analyzed a set of widely used processing parameters that have a significant influence on this process. Using the deduced dimensionless variables, the authors applied the aforementioned process and proved that the variables could describe and predict the SLM process phenomenon for continuous track forming, track size, and porosity evolution.

Starting from the standpoint of metal 3D printing complexity as well as its complicated process optimization, materials development, real-time monitoring, and control problems, the authors of [43] performed useful dimensional analysis. In their study, based on ultra-high-speed synchrotron X-ray imaging and high-fidelity multi-physics modeling, they proposed simple and universal scaling laws for keyhole stability and porosity in metal 3D printing.

The laws are broadly applicable and remain accurate for different materials, processing conditions, and printing machines. The deduced compact scaling laws contribute both to process optimization and defect elimination during printing and also to a quantitative predictive framework. It is a well-known fact that 3D metal printing incorporates numerous parameters with complex interactions and dependencies, which must be taken into consideration when manufacturing a given component.

Nowadays, there are no universal physical relationships that can be applied to different materials, machines, or processing conditions. In the case of laser powder bed fusion, due to vaporization-induced recoil pressure, a topological depression frequently appears, known as a keyhole. Its dynamics are very difficult to understand and predict as they depend on several physical mechanisms; however, it is closely related to energy absorption and defect formation in metal 3D printing. A keyhole’s geometry strongly influences the energy coupling mechanism between a high-power laser and the material being used which consequently produces unusual melt pool dynamics and solidification defects. In addition, an unstable keyhole produces severe process instability and structural defects such as porosity, the balling effect, spattering, and unusual micro-structural phases.

Previous research capturing meso-nanosecond keyhole dynamics with high-fidelity simulations produced evidence of keyhole-induced back-spattering and frozen depression defects [59]. The authors conducted high-energy X-ray imaging experiments in the context of laser melting of bare plates, powder beds, and powder flow. Based on in situ X-ray analysis, the authors investigated the effect of process conditions on the keyhole’s behavior in the context of laser melting to austenitic stainless steel. They found that with an increase in laser power, the depth and width of a keyhole increase, while fluctuations in its depth decrease. In addition, in the absence of a shielding gas, a large fluctuation in keyhole depth produces an attenuation of the laser power, as well as changes in laser beam quality, due to the interaction between the laser and the metal vapor. The authors observed that together with the laser’s traversal speed increasing with respect to the keyhole-forming speed, the keyhole’s inclination decreased, while the width and opening of the keyhole expanded. The authors also proposed some useful dimensionless variables that help to improve the quality and efficiency of the laser-melting process regarding keyhole formation in procedures such as AM and laser welding and cutting.

In the context of metal thin-walled component production (of prototypes or small series components), AM technologies have several limitations, such as size, price, productivity, and the type of requested materials [55]. At the same time, conventional manufacturing methods also have significant limitations related to the product’s shape, which usually correlates with the optimization methods used (topological optimization or generative design). Consequently, hybrid technologies, which combine the advantages of AM and conventional technologies, represent the optimal solution. The authors of [55] describe the application of topological optimization combined with technological co-design to improve the design of aluminum casting methodology. The requirements of hybrid technology were analyzed for this material, as well as the model’s correlation with the production and operations of investment casting technology. Based on an accurate optical roughness measurement, they compared the surface quality of a standard wax model with that of a model obtained with AM from a given polymer using the binder jet method. The authors obtained a better-quality product with the latter technique; however, there were additional requirements for prototype production technology. Furthermore, using precise measurements, the same authors proved that this polymer model had half the magnitude of thermal expansion in the measured range compared with the wax model, which represents minimal shape deviation; this finding was also confirmed using dimensional analysis. It is well known that during the metal additive manufacturing process, e.g., with the Inconel 718 (IN718) alloy, manufacturing defects and micro-structural heterogeneities will appear, as well as the micro-segregation of secondary Laves phases, which are major issues that need to be resolved. Thus, the authors applied annealing with air cooling, followed by double aging with furnace cooling.

The authors of [57] performed a useful dimensional analysis study on the samples obtained with laser-based power bed fusion additive technology using the IN718 alloy in order to study the effect of thermal loading and double-aging heat treatment. They found that following the heat treatment at the micron scale, the volumetric shrinkage of the tested specimens was 3.084%. In the same time period, the hardness of the heat-treated samples increased to 35% and showed an increase in hardness from 417 to 564 HV.

The authors of [8] conducted a search and investigation into a comparative analysis of two widely used polymers, i.e., ABS and PLA, with two metal-infused thermoplastics, i.e., copper-enhanced PLA and aluminum-enhanced ASA (acrylonitrile styrene acrylate). They started their investigations because the traditional FFF method is a cost-effective AM that unfortunately has several limitations, such as high levels of roughness, poor mechanical properties, and selective dependence on the material. In contrast, metal-infused thermoplastics offer better properties. It should be noted that the properties of FFF components can be improved with post-processing. All four types of samples were subjected to post-processing (i.e., annealing) and were subsequently investigated using ultrasonic testing, hardness tests, tensile tests, and microstructural analyses as well as using dimensional analysis to highlight the annealing effect differently. It was evident that for semi-crystalline materials (i.e., PLA and copper-enhanced PLA), the tensile strength increased significantly due to annealing. However, in the case of amorphous materials (ABS and aluminum-enhanced ASA), this effect was much smaller, probably due to the longer transmission times and a high percentage of voids. In addition, the maximum hardness corresponded to ASA, while the lower hardness corresponded to ABS material. Using the obtained results, the authors provided a good delimitation of the two processes while also demonstrating their applicability with commonly used PLA and ABS materials.

At this point, we should mention that the objective of this review was not to analyze the topic of conventional mathematical modeling. Our research only focused on the dimensional methods involved in AM methods and the improvement of its products.

In the next section, we offer a brief discussion of our results by applying the advantages of MDA.

Although we only performed analyses on some simple specimens, using the deduced MLs, it was possible to provide evidence of the flexibility and simplicity of MDA, which will be important in the future for the many common engineers involved in AM, offering an efficient, unitary, and user-friendly approach.

## 3. Engineering Applications for MDA

At first glance, some readers may question whether it is necessary to include engineering applications for MDA in this paper. However, in our opinion, the inclusion of MDA is perfectly justified, serving to systematically illustrate its effectiveness.

Similar approaches have been used to illustrate the analyzed issues, which appear frequently in the specialized literature cited above [15,176]. In addition, based on their meticulous evaluations of the proposed topics, several authors considered it useful to illustrate the synthesized phenomenon through their praxis.

In this section, we illustrate the advantages of MDA compared with other dimensional methods. At the same time, we show how AM technologies can be made more efficient using MDA, which is a very flexible, simple, and easy-to-use procedure compared with the other dimensionless procedures analyzed above.

Among other things, the model that is attached to the prototype can be made very manageable, which ensures not only the cost-effectiveness of the manufacturing process (both for the model and prototype) but also enables highly efficient, simple, and repeatable experimental investigations to be performed on the model (see the advantages of *MDA* presented in the Introduction).

In the following section, we illustrate the main advantages of MDA based on our previous contribution to the literature [152]. To this end, we analyze how appropriate MLs, which are suitable for different components, can be deduced and validated, and then follow the same process for components manufactured from polymers using classical AM technology. The main goal of our research was to obtain an optimal structure with maximal stiffness while at the same time ensuring the minimal consumption of materials [152].

This objective, which was achieved using a double-level ribbed cross-section, offered a good opportunity to incorporate the filling parameter in the ML as an independent variable. Consequently, it became possible to propose several useful filling methods for both the prototype and the model. This independent variable not only provides a desired filling percentage of the nominal volume but also lends the desired stiffness for the prototype respective to the model.

**Figure 1:**
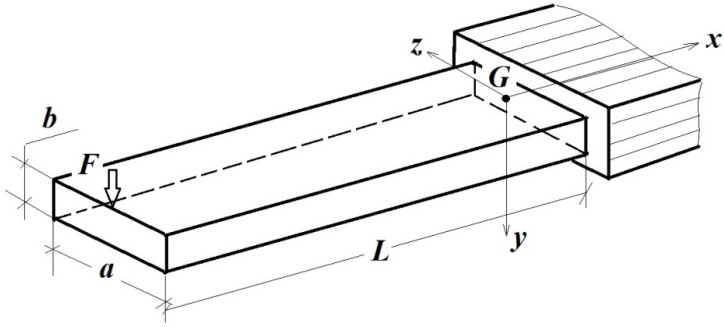
The tested beam [152].

To validate the independent variable, we designed a simple structure incorporating a simple cantilever beam that was subjected to bending, as shown in Figure 1 [152]. The dimensions a [m], b[m], L[m], the reference system xGyz, and the applied force F[N] are provided.

**Figure 2:**
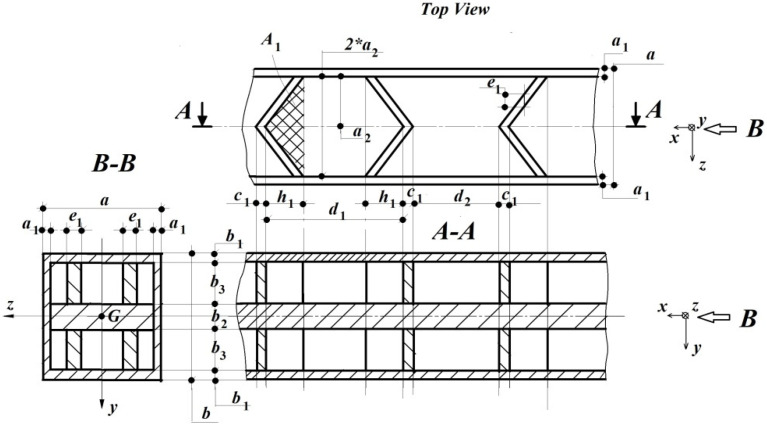
The proposed two-level filling version of the beam’s volume [152].

Figure 2 presents a possible, rib-based, filling scenario for the beam’s volume.

By incorporating this approach, it was possible to attain the well-known and often preferable honeycomb structure, which showed a very good correlation with the involved material and its strength behaviors. As mentioned in Section 2, all the parameters related to the prototype were indexed with (1), while for the model indexing, (2) was used.

In order to illustrate the capabilities of the applied MDA, the main steps of this approach are briefly presented as follows:

a The possible influencing variables of the analyzed phenomenon were chosen for the $v_{1}\left[m\right]$ magnitude of the prototype’s vertical displacement at its free end.

These variables are as follows:
  - The beam dimensions of a[m],b[m],L[m], as well as the $A_{1}\left[m^{2}\right]=\frac{2\cdot a_{2}\cdot h_{1}}{2}=a_{2}\cdot h_{1}$ area defined by the ribs;
  - The applied F[N] force;
  - The $E\left[N/m^{2}\right]$, Young’s modulus;
  - The $V_{util}\left[m^{3}\right]$ calculation for the useful volume of the beam, which is related to the filling degree.

In addition, by combining and margining these variables, they offer several useful modalities for obtaining a more flexible model associated with the given prototype, which will be analyzed in the following paragraphs.

Therefore, we analyzed the following:
  - The $I_{z}\left[m^{4}\right]$ second-order moment of inertia, instead of the given a[m],b[m] cross-sectional dimensions;
  - The involvement of the $E\cdot I_{z}\left[N\cdot m^{2}\right]$ stiffness module, if necessary, along with the density $\rho \left[\frac{N\cdot s^{2}}{m^{4}}\right]$ or specific gravity $\gamma \left[\frac{N}{m^{3}}\right]$.

b The definition of matrix *A*, including the exponents of the involved dimensions of the independent variables, were calculated. Following the method detailed in Section 2, these variables can be freely chosen a priori for both the prototype and the model. This matrix must be invertible, i.e., det|A|≠0.

c The exponents of the remaining variables formed matrix *B*. These variables are the so-called dependent variables, which can only be freely chosen a priori for the prototype. This category also included the desired $v_{1}\left[m\right]$ vertical displacement of the prototype, which we assumed would be difficult to obtain using direct experimentation on the prototype. Consequently, it was determined using one element of the *ML*.

d The set of matrices designated *B-A* was completed with the matrices $C=-{\left(A^{-1}\cdot B\right)}^{T},$ respectively, to achieve $D\equiv I_{nxn}$, which, together with matrices *A* and *B*, constitute the dimensional set in the form shown in Table 1.

In the above-mentioned relationship, $D\equiv I_{nxn}$ is an n⋅n unit matrix, $\left(A^{-1}\right)$ represents the inverse of the matrix *A*, and ${\left(A^{-1}\cdot B\right)}^{T}$ represents the transposition of the product $\left(A^{-1}\cdot B\right).$

e Based on the unique protocol deduced in [149,150], the elements of the desired *ML* were obtained, where, instead of the given/included $\omega_{j},\;j=1,\ldots ,n$ variables are placed, along with their corresponding scale factors:

$$S_{\omega_{j}}=\frac{\omega_{j,2}}{\omega_{j,1}}.$$

The authors of [152] analyzed three remarkable variants of the dimensional set, which are presented in the following paragraphs, together with their facilities and limits.

a. Using the independent variables $E\left[N/m^{2}\right]$ and $I_{z}\left[m^{4}\right]$, one can demonstrate the significant fact that the remaining parameters, $a^{**}\left[m\right]\Leftrightarrow a_{1},\;a_{2},\;b_{1},\;b_{2},\;b_{3},\;c_{1},\;d_{1},\;d_{2},\;e_{1},\;h_{1}$ can be divided into others such as $a^{*}\left[m\right]\Leftrightarrow a_{1},\;a_{2},\;e_{1},$; $b^{*}\left[m\right]\Leftrightarrow b_{1},\;b_{2},\;b_{3}$; and $c^{*}\left[m\right]\Leftrightarrow c_{1},\;d_{1},\;d_{2},\;h_{1}$, without affecting the final ML. In addition, for the correct merging of the initial sizes a[m], b[m] in $I_{z}\left[m^{4}\right]$, a and b can no longer appear in other elements as they are used here in $a^{*},\;b^{*},\;c^{*}$. Table 2 shows the corresponding dimensional set.

To illustrate the unique protocol mentioned above, the following calculations were made.

From the first line, comprising $\pi_{1}$, the exponents of the variables led to the following product, which is equal to unity. Subsequently, the variables were substituted with their scale factors, finally resulting in the first ML:

(1) $$\pi_{1}=v\cdot E^{0}\cdot I_{z}^{-0.25}=1\Rightarrow S_{v}=\sqrt[{4}]{S_{I_{z}}}.$$

In this relationship (1), based on the experimental measurements made on the model, the size $v_{2}$ is known. Consequently, $S_{v}=\sqrt[{4}]{S_{I_{z}}}=\frac{v_{2}}{v_{1}}$ will finally resulted in the size of the predictable $v_{1}$, that is,

(2) $$v_{1}=\frac{v_{2}}{\sqrt[{4}]{S_{I_{z}}}}$$

In a similar manner, the rest of the elements of the *ML* were analyzed. In the first approach, the size of the force $F_{2}$ applied to the model is important since this identifies the specific amount of force that would be required for the prototype:

(3) $$\pi_{2}=F\cdot E^{-1}\cdot I_{z}^{-0.5}=1\Rightarrow S_{F}=S_{E}\cdot \sqrt{S_{I_{z}}};$$

so,

(4) $$F_{2}=F_{1}\cdot S_{E}\cdot \sqrt{S_{I_{z}}};\mathrm{etc}.$$

b. In the second version, by merging $E\left[N/m^{2}\right]$ and $I_{z}\left[m^{4}\right]$ into the new independent variable of the stiffness modulus $E\cdot I_{z}\left[N\cdot m^{2}\right]$, it was possible to introduce one other independent variable: the beam L[m] length. Consequently, it was possible to freely choose the length of the prototype and the length of the model became possible.

In addition, one had the opportunity to make a more favorable choice of material type in combination with an equally preferable cross-section.

The corresponding dimensional set is presented in Table 3 [152].

In this case, by applying the above protocol, the seven elements of the ML were obtained:

(5) $$\pi_{1}=v\cdot L^{-1}\cdot {\left(E\cdot I_{z}\right)}^{0}=1\;\Rightarrow \;S_{v}=S_{L};$$

(6) $$\pi_{2}=a*\cdot L^{-1}\cdot {\left(E\cdot I_{z}\right)}^{0}=1\;\Rightarrow \;S_{a*}=S_{L};$$

(7) $$\pi_{3}=b*\cdot L^{-1}\cdot {\left(E\cdot I_{z}\right)}^{0}=1\;\Rightarrow \;S_{b*}=S_{L};$$

(8) $$\pi_{4}=c*\cdot L^{-1}\cdot {\left(E\cdot I_{z}\right)}^{0}=1\;\Rightarrow \;S_{c*}=S_{L};$$

(9) $$\pi_{5}=A_{1}\cdot L^{-2}\cdot {\left(E\cdot I_{z}\right)}^{0}=1\;\Rightarrow \;S_{A_{1}}={\left(S_{L}\right)}^{2};$$

(10) $$\pi_{6}=F\cdot L^{3}\cdot {\left(E\cdot I_{z}\right)}^{-1}=1\;\Rightarrow \;S_{F}=\frac{S_{EIz}}{{\left(S_{L}\right)}^{3}};$$

(11) $$\pi_{7}=V_{u}\cdot L^{-3}\cdot {\left(E\cdot I_{z}\right)}^{0}=1\;\Rightarrow \;S_{V_{u}}={\left(S_{L}\right)}^{3}.$$

Of these, for concrete applications, those related to $v_{1}$ and $F_{2}$ were of particular interest, i.e., relationships (5) and (10).

c. In the third approach, the useful volume $V_{util}\left[m^{3}\right]$ (which evaluates the degree of the filling) and the $E\cdot I_{z}\left[N\cdot m^{2}\right]$ stiffness modulus were accepted as independent variables. In this case, the beams lengths and the applied forces were dependent variables with easy applicability in the prototype and the model. Table 4 shows the corresponding dimensional set [152].

From the deduced *ML*, only three elements are presented, namely, those related to the vertical displacement of the free end of the prototype $V_{1}$, the force that must be applied to the model $F_{2}$, and the required length of the model $L_{2}$:

(12) $$\pi_{1}=v\cdot V_{u}^{-0.333}\cdot {\left(E\cdot I_{z}\right)}^{0}=\frac{v}{\sqrt[{3}]{V_{u}}}=1\;\Rightarrow \;S_{v}=\sqrt[{3}]{S_{V_{u}}}.$$

(13) $$\pi_{6}=F\cdot V_{u}^{0.666}\cdot {\left(E\cdot I_{z}\right)}^{-1}=\frac{F\cdot \sqrt[{3}]{V_{u}^{2}}}{E\cdot I_{z}}=1\;\Rightarrow S_{F}=\frac{S_{EI_{z}}}{\sqrt[{3}]{{\left(S_{V_{u}}\right)}^{2}}}$$

(14) $$\pi_{7}=L\cdot V_{u}^{-0.333}\cdot {\left(E\cdot I_{z}\right)}^{0}=\frac{L}{\sqrt[{3}]{V_{u}}}=1\;\Rightarrow \;S_{L}=\sqrt[{3}]{S_{V_{u}}}.$$

To illustrate the deduced MLs, we chose the second variant, where the value deduced using Equation (5) and the experimentally obtained $V_{1}$ for the displacement of the free end on the prototype are compared.

For this reason, we utilized solid rectangular cross-sections for both the prototype and the model, manufactured from the same common PLA (polylactic acid) material, where $E_{1}=E_{2}=2.31\times 10^{9}\;\frac{N}{m^{2}}$.

The prototype had a length of $L_{1}=0.400\;m$, a cross-section of 0.018 × 0.030 m, and was subjected to a force of $F_{1}=0.491\;N$. The attached model had a length of $L_{2}=0.300\;m$ and a cross-section of 0.010×0.020 m.

The corresponding axial moments of inertia were as follows:

(15) $$I_{z_{1}}=\frac{0.018\cdot {\left(0.030\right)}^{3}}{12}=40.05\cdot 10^{-9}\;m^{4};I_{z_{2}}=\frac{0.010\cdot {\left(0.020\right)}^{3}}{12}=6.67\cdot 10^{-9}\;m^{4}.$$

The resulting scale factors were $S_{E\cdot I_{z}}=S_{I_{z}}=0.167$ at $S_{L}=\frac{L_{2}}{L_{1}}=0.75$.

Consequently, from Equation (10), we obtained the following:

(16) $$S_{F}=\frac{S_{EIz}}{{\left(S_{L}\right)}^{3}}=\frac{F_{2}}{F_{1}}\;\Rightarrow \;F_{2}=F_{1}\cdot \frac{S_{EIz}}{{\left(S_{L}\right)}^{3}}=0.491\cdot \frac{0.167}{{\left(0.75\right)}^{3}}=0.203\;N$$

Using metrological accuracy measurements on the model, a force of $F_{2}$ resulted in a displacement of $v_{2}=0.000583\;m$, while from the first element of the ML, represented by Equation (5), we obtained:

(17) $$S_{v}=S_{L}=\frac{v_{2}}{v_{1}}\Rightarrow \;v_{1}=\frac{v_{2}}{S_{L}}=\frac{0.000583}{0.75}=0.000778\;m.$$

After carrying out actual measurements on the prototype, we obtained a value of $v_{1\;M}=0.000786\;m$, representing an error of 1.08%, which is acceptable from an engineering point of view.

Consequently, the ML deduced using the second version is fully validated.

In a similar manner, using the corresponding independent variables, the first and the third variants are also validated.

In the first variant, one can choose different materials, e.g., different kinds of polymers, a given polymer and a given metal, or two different kinds of metals.

In the third version, there are more opportunities for variation including choosing different materials and/or different cross-sectional sizes and shapes and choosing different filling percentages.

## 4. Conclusions

It should be noted that combining AM with the generally accepted casting technologies offers several advantages and improves casting flexibility. This method obtains more complex products and also optimizes their manufacturing process (lowering preparation costs, shortening manufacturing time, increasing accuracy and durability, and reducing the amount of waste produced).

Manufacturing casting molds or cores using AM, especially when combining metal and plastic layers, represents a possible direction for future research. In this context, several common problems can be resolved in the final product, i.e., a mold, such as its accuracy, surface quality, strength against mechanical and thermal loading, dimensional stability, durability, and lifespan.

The following conclusions can be formulated:

- It should be emphasized that the deduced MLs cannot represent all physical relationships in the truest sense; they can only provide useful correlations between the involved scale factors of the variables. This helps us to obtain definite correlations between the relative behavior of the prototype and model.
- As previously illustrated, MDA offers great flexibility when selecting a suitable (best-fit) model since it can be both manufactured and tested under optimal conditions. This allows for inexpensive preparation, experimental testing, the need for fewer qualified persons, etc.
- We only tested the efficiency of MDA for specimen optimization. However, this approach, using the dimensional analysis conceptual modeling (DACM) framework developed by Coatanéa [157,158,159,160,161,162,163,164,165,166,167], can also be extended and successfully implemented in different areas of additive manufacturing.
- The MLs we deduced are also fully applicable to metals.
- Our future goal is to design and validate suitable *MLs* for combined metal–polymer components, as well as for components with different internal structures.

## Figures and Tables

**Table 1:** The dimensional set [149,150].

***B***	***A***
***D***	***C***

**Table 2:** The dimensional set of the first version [152].

Dimensions	B								A	
	v	F	L	a*	b*	c*	A_1_	V_util_	E	I_z_
m	1	0	1	1	1	1	2	3	−2	4
N	0	1	0	0	0	0	0	0	1	0
π1	1	0	0	0	0	0	0	0	0	−0.25
π2	0	1	0	0	0	0	0	0	−1	−0.5
π3	0	0	1	0	0	0	0	0	0	−0.25
π4	0	0	0	1	0	0	0	0	0	−0.25
π5	0	0	0	0	1	0	0	0	0	−0.25
π6	0	0	0	0	0	1	0	0	0	−0.25
π7	0	0	0	0	0	0	1	0	0	−0.5
π8	0	0	0	0	0	0	0	1	0	−0.75

**Table 3:** The dimensional set of the second variant [152].

Dimensions	B							A	
	v	a*	b*	c*	A_1_	F	V_util_	L	E*I_z_
m	1	1	1	1	2	0	3	1	2
N	0	0	0	0	0	1	0	0	1
π1	1	0	0	0	0	0	0	−1	0
π2	0	1	0	0	0	0	0	−1	0
π3	0	0	1	0	0	0	0	−1	0
π4	0	0	0	1	0	0	0	−1	0
π5	0	0	0	0	1	0	0	−2	0
π6	0	0	0	0	0	1	0	3	−1
π7	0	0	0	0	0	0	1	−3	0

**Table 4:** The dimensional set of the third variant [152].

Dimensions	B							A	
	v	a*	b*	c*	A_1_	F	L	V_util_	E*I_z_
m	1	1	1	1	2	0	1	3	2
N	0	0	0	0	0	1	0	0	1
π1	1	0	0	0	0	0	0	−0.33333	0
π2	0	1	0	0	0	0	0	−0.33333	0
π3	0	0	1	0	0	0	0	−0.33333	0
π4	0	0	0	1	0	0	0	−0.33333	0
π5	0	0	0	0	1	0	0	−0.66667	0
π6	0	0	0	0	0	1	0	0.666667	−1
π7	0	0	0	0	0	0	1	−0.33333	0

## Data Availability

Not applicable.
